# Editorials

**Published:** 1890-07

**Authors:** 


### HOMŒOPATHIC PHYSICIAN,

A MONTHLY JOURNAL OF

HOMŒOPATHIC MATERIA MEDICA AND CLINICAL MEDICINE.

If our school ever give up the strict inductive method of Hahnemann, we are lost, and deserve only to be mentioned as a caricature in the history of medicine."—CONSTANTINE HERING.

Vol. X. JULY, 1890. No. 7.

### EDITORIALS.

### THE USE OF CRUDE DRUGS BY PROFESSED HOMŒOPATHS.

"Hast thou not spoke like thunder on my side?
Been sworn my soldier? bidding me depend
Upon thy stars, thy fortune, and thy strength?
And dost thou now fall over to my foes?
Thou wear'st a lion's hide! doff it for shame,
And hang a calf's skin on those recreant limbs."

If nothing else served to remind us of the presence of mongrels, we have the agents of various drug manufacturers, who frequently call on us and wish to leave samples of their several compounds, and who, on being told that we are homœopathicians, and do not use such preparations, always reply: "Why, homœopathic physicians do use them, and we sell to almost all we call upon." We think we convince them to the contrary before they leave. We simply point out the difference between honesty and dishonesty by showing them that he who claims to be a homœopathist cannot conscientiously use their goods. It requires but a moment's conversation to have them agree with us, and they always express contempt for those who forswear Homœopathy by buying, and—in many instances clandestinely—using these compounds of crude drugs.

Genuine Homœopathy suffers more at the hands of its pretended friends than from any other one cause. The intolerance and bigotry of allopathy have done more good to our cause than harm. All the bleating and quacking of those who have tried to put it down by argument and abuse have only been to the interest of our school, and thereby been the means of making us better known. Thus, many precious lives have been saved that otherwise would have been sacrificed to the lawless empiricism of allopathy. Unfortunately for many sick people, and for other many who are in health, there is a large class of unprincipled men who are trading on the good name of Homœopathy. There are many of this class who are so ignorant of what constitutes Homœopathy that it is but charity to say that they "know not what they do;" therefore, it is but justice to add that they are not responsible. But by far the larger number are engaged in what they call practicing medicine for the purpose of making money only. One of the latter lately said to us: "I find that I can make more money by being known as a homœopath than by any other means." And the rascal was using means that would put the worst drugger in the allopathic ranks to shame.

Many of these men do know what Homœopathy is and what it can do for the sick, but they acknowledge that it is much easier to give a crude drug that will have an immediate, visible effect, and thus have the patient believe he is being cured, than to spend time and effort—which they know to be necessary—in finding the remedy which is demanded by our law of therapeutics.

This we know to be the cause of the apostacy of a large number who have no excuse to offer for their betrayal of a cause which, when honestly adhered to, is capable of so much good. It is through the work of these pretenders that we often hear it said: "Homœopathic doctors give medicine just as strong, and use as much Morphia and Quinine as the old school use." Of course, those who know an honest practitioner of Homœopathy know better than this, but those who do not know are the ones whom we must enlighten. Therefore, in order that our good cause may not be made to suffer, let us "be instant in season and out of season." Let us never hesitate to show to those who do not know that the man who takes the name of homœopathist, and who uses crude drugs as an old-school doctor would use them is but trading on the name, and that he is "living a lie." We were willing to resort to the use of crude drugs if only some one would answer for us this question :

> "What rhubarb, senna, or what purgative drug
> Would scour these *men* hence? Hearest thou of them?"

G. H. C.

---

"THE REPETITION OF THE DOSE of a medicine is regulated by the duration of the action of each medicine. If the remedy acts in a positive (curative) manner, the amendment is still perceptible after the duration of its action has expired, and then another dose of the suitable remedy destroys the remainder of the disease. The good work will not be interrupted if the second dose be not given before the lapse of some hours after the cessation of the action of the remedy. The portion of the disease already annihilated cannot in the meantime be renewed, and even should we leave the patient several days without medicine, the amelioration resulting from the first dose of the curative medicine will always remain manifest.

"So far from the good effect being delayed by not repeating the dose until after the medicine has exhausted its action, the cure may, on the contrary, be frustrated by its too rapid repetition. For this reason, because a dose prescribed before the cessation of the term of action of the positive medicine is to be regarded as an augmentation of the first dose, which, from ignorance of this circumstance, may thereby be increased to an enormous degree, aud then prove hurtful by reason of its excess.

"I have already stated that the smallest possible dose of a positively acting medicine will suffice to produce its full effect. If, in the case of a medicine whose action lasts a long time, as, for instance, digitalis, where it continues to the seventh day, the dose be repeated frequently, that is to say three or four times in the course of a day, the actual quantity of medicine will, before the seven days have expired, have increased twenty or thirty fold, and thereby become extremely violent and injurious; whereas the first dose (a twentieth or thirtieth part) would have amply sufficed to effect a cure without any bad consequences.*

"After the expiring of the term of action of the first dose of the medicine employed in a curative manner, we judge whether it will be useful to give a second dose of the same remedy. If the disease have diminished in almost its whole extent, not merely in the first half-hour after taking the medicine, but later, and during the whole duration of the action of the first dose, if this diminution have increased all the more, the nearer the period of the action of the remedy approached its termination—or even if, as happens in very chronic diseases, or in maladies, the return of whose paroxysm could not have been expected during this time, no perceptible amelioration of the disease have indeed occurred, but yet no new symptom of importance, no hitherto unfelt suffering deserving of attention have appeared, then it is in the former case almost invariably certain, and in the latter highly probable, that the medicine was the curatively helpful, the positively appropriate one, and, if requisite, ought to be followed up by a second; and, finally, even after the favorable termination of the action of the second by a third dose if it be necessary, and the disease be not in the meantime completely cured, as it often is, in the case of acute diseases, by the very first dose."—*The Medicine of Experience, Hahnemann*, 1805.

---

* The following circumstance must also be taken into consideration. We cannot well tell how it happens, but it is not the less true, that even one and the same dose of medicine, which would suffice for the cure, provided it were not repeated before the action of the remedy had ceased—acts ten times as powerfully, if the dose be divided, and these portions taken at short intervals during the continuance of the action of the medicine. For example, if the dose of ten drops, which would have sufficed for the cure, be divided among the five days during which the action of medicine lasts in such a manner as that one drop of it shall be taken twice a day, at the end of five days the same effect is not produced as would have occurred from ten drops given at once every five days, but a far more powerful, excessive, violent [cumulative] effect, provided that the medicine was a curative and positive antidote to the disease.

The question which Hahnemann treats in the above paragraphs is one that should be constantly before every physician. Although it has been written about and much discussed since Hahnemann's day, nothing new or better has been added to it. Its importance cannot be overrated. How little attention is given it in so-called homœopathic colleges can readily be learned from any recent graduate. It is so essential to the proper management of every case of sickness that it should be indelibly impressed upon the memory of every one who professes to have a knowledge of Homœopathy. Without it no case can be intelligently treated.

The choice of the curative is at times comparatively easy. But when to give the second dose and when not to give are the momentous questions.

By following the plain instructions given by Hahnemann no one can go astray. This clear advice is not based upon any theory. It is the result of years of observation by a man whose genius is known—the Sage of Cœthen.

This counsel should be more closely adhered to in treating children and those who are readily affected by remedies; more particularly in paroxysmal affections. It should be our invariable rule never to repeat the first dose so long as the condition which is described above by Hahnemann obtains. Thus, in intermittent affections only give the dose between the paroxysms; never during the attack—preferably immediately after. Then adhere to the guide and the best results will be obtained. The same applies to diarrhœa. If after the first dose—which should be given immediately after an evacuation—there be an improvement, give no more medicine so long as the improvement continues. Convulsions, spasmodic affections, and all maladies of a remittent character come under this rule.

Cleave to this. Watch the results. Then, if you have not been practicing with this constantly before you, compare your success with your previous practice. G. H. C.

---

Dr. Guernsey's Answer to the correspondence of Professor Edmund Carleton, given in our last number, appears this month at page 323. It constitutes another chapter in the famous Ward's Island controversy.

